# Editorial: Pharmacoepidemiology in chronic diseases

**DOI:** 10.3389/fepid.2026.1963125

**Published:** 2026-10-30

**Authors:** Minji Sohn

**Affiliations:** College of Pharmacy, Sahmyook University, Seoul, Republic of Korea

**Keywords:** chronic disease, drug safety, drug utilization, medication adherence, pharmacoepidemiology, pharmacovigilance, polypharmacy, real-world evidence

The long-term management of chronic diseases accounts for a substantial proportion of medicine use. However, patients treated in routine care differ from those enrolled in trials that support regulatory approval. These real-world populations are often older and more likely to have multimorbidity, polypharmacy, and treatment durations that extend beyond the follow-up period of clinical trials. Across healthcare systems, treatment is influenced by reimbursement policies, formulary restrictions, and access barriers. Pharmacoepidemiology addresses this evidence gap by describing how medicines are used in practice and estimating their real-world effectiveness and safety, thereby informing prescribing practices, regulatory labeling, and health policies (1, 2).

This Research Topic integrates observational, pharmacovigilance, and health-policy studies spanning the continuum of chronic disease care. The call for papers solicited studies on medicine effectiveness and safety, drug utilization, patient safety, post-marketing surveillance, pharmacoeconomics, and methodology. This Research Topic captures each of these domains. Several contributions further address the demanding questions identified in the original call that remain challenging to examine in routine care, including drug interactions, exposure–response relationships, genetic determinants of treatment response, and nonadherence. Across 27 articles, the contributing studies draw on diverse sources, including national insurance, administrative, and vital statistics databases from Taiwan, Sweden, Italy, China, and the United States; spontaneous reporting systems from the United States and Japan; hospital-based cohorts from Qatar, China, and Spain; and survey and dispensing data from Jordan, Colombia, and Uruguay. Collectively, these studies converge on five key themes, providing critical insights into the interpretation of real-world evidence for chronic disease management.

## Safety signal detection in long-term pharmacotherapy

Nine articles used spontaneous adverse event reporting databases to characterize the safety of long-term pharmacotherapy for chronic diseases. All studies used the US Food and Drug Administration Adverse Event Reporting System (FAERS), and one (Zhang et al.) extended the analysis to a second system, the Japanese Adverse Drug Event Report database (JADER). This concentration reflects the scale and open accessibility of FAERS, but also means that most of the signals described below are conditioned on reporting behavior in a single regulatory setting. Since a disproportionality estimate reflects the reporting process as much as the drug itself (3), replication in JADER, EudraVigilance, or the World Health Organization VigiBase is one of the clearest ways to distinguish drug-related patterns from reporting artifacts.

Several studies examined immunomodulatory therapies that are often administered for years, rather than weeks. Xiong et al. analyzed malignancy reports associated with disease-modifying anti-rheumatic drugs (DMARDs) in rheumatoid arthritis and detected signals for a broader range of malignancies with targeted synthetic DMARDs than with other DMARDs. Drug-specific median times to onset ranged from 0.83 to 1.67 years for targeted synthetic agents and from 3.58 to 7.08 years for conventional synthetic agents. Yu et al. focused on older adults receiving tumor necrosis factor-α inhibitors and found substantial heterogeneity among agents, with etanercept showing the weakest and certolizumab pegol showing the strongest overall infection signals, while golimumab showed the strongest signal for tuberculosis and the highest reporting odds among patients with ankylosing spondylitis. Liu et al. assessed the interleukin-1 blockers anakinra and canakinumab and found profiles broadly consistent with existing labeling. Injection-site reactions were more frequent with anakinra, whereas gastrointestinal and respiratory events were reported more often with canakinumab and occurred predominantly in children and adolescents. Neither drug showed a significant signal for tuberculosis.

Other studies applied this approach to newer or less well-studied exposures. Zhang et al. characterized the post-marketing profile of onasemnogene abeparvovec in spinal muscular atrophy. Most events were reported early, particularly within the first 8 days after administration, and thrombocytopenia and thrombotic microangiopathy were more frequently reported in patients weighing at least 8.5 kg. Zhang et al. combined FAERS with JADER to quantify reports of serotonin syndrome associated with the concomitant use of opioids and serotonergic antidepressants, a clinically relevant combination in perioperative care. Zhu et al. paired a disproportionality analysis of drug-induced gingival overgrowth with network pharmacology and identified reporting signals involving bisphosphonates, in addition to the three drug classes traditionally implicated: calcium channel blockers, immunosuppressants, and antiepileptics. Chen et al. prespecified analyses of cardiovascular events reported with tirzepatide, stratified by reporter type, given that consumer submissions accounted for 92.3% of all reports. In analyses restricted to reports from healthcare professionals, tirzepatide had lower reporting odds for heart failure than all other drugs in the concurrent FAERS comparator (reporting odds ratio [ROR] 0.18, 95% confidence interval [CI] 0.07–0.43), with no reporting signal for atrial fibrillation or tachycardia. Elevated reporting odds for tachycardia were observed only in weight-stratified analyses of the full dataset.

These studies illustrate both the utility and limitations of disproportionality analysis. It is rapid, draws on reports from broad post-marketing populations, and can detect signals for events that are too rare to characterize adequately in pre-approval trials. It also lacks a denominator, cannot estimate incidence, and remains highly sensitive to reporting behavior, notoriety bias, confounding by indication, and channeling bias (4). Although studies based on a single spontaneous reporting system rarely provide definitive evidence on their own, they remain valuable when they address clinically important questions, probe rare or delayed events that are difficult to capture in trials, or incorporate design features that sharpen interpretation.

Three articles showed how these limitations can be addressed through study design rather than merely being acknowledged. Chen et al. stratified by reporter type, whereas Spadotto et al. addressed the long-standing question of whether ivabradine causes atrial fibrillation by combining a meta-analysis of randomized trials with a disproportionality analysis using beta-blockers as active comparators. In randomized trials, the ratio of ischemic cerebrovascular events to atrial fibrillation was lower with ivabradine than with placebo. This finding is consistent with the enhanced detection of atrial fibrillation, although a direct effect on incidence cannot be excluded. The authors present this detection explanation as a hypothesis requiring prospective evaluation. Tan et al. used a comparable dual strategy for calcitonin analogs in osteoporosis in older adults, pairing signal detection with a meta-analysis of randomized controlled trials that found no significant effect on fracture prevention.

## Medicine exposure and outcomes in population-based studies

The second thematic group of studies relied on population-based observational data to examine the association between medicine exposure and clinical outcomes. In such designs, the definition and timing of exposure relative to the follow-up period can profoundly impact effect estimates (5). Several studies mitigated these methodological risks by employing cumulative dose stratification, latency-sensitive analyses, or active comparator designs to minimize selection and confounding biases.

Li et al. drew on Taiwan's National Health Insurance Database covering the period 2008–2022 to examine the association between dipeptidyl peptidase-4 inhibitor use and diabetic retinopathy. They found a graded association across the categories of cumulative defined daily doses (DDD) relative to propensity score-matched sulfonylurea users. Yu et al. addressed the long-contested question of the association between antihypertensive medicine use and cancer in 270,320 patients with newly diagnosed hypertension and found no clinically meaningful overall association. The marginal signal for calcium channel blockers did not persist in analyses that excluded early follow-up periods, illustrating how strongly such estimates depended on the latency assumptions. Bérard et al. examined the pregnancy outcomes associated with exposure to dihydroergotamine or triptans in 767,994 pregnancies. After adjusting for maternal migraines, drug exposure was not significantly associated with preterm birth, low birth weight, major congenital malformations, or spontaneous abortion. The estimates based on 189 dihydroergotamine-exposed pregnancies were highly imprecise. The authors regarded the observed association with septal defects as a chance finding requiring replication.

Jung et al. used complementary clinical and epidemiologic approaches. They described the clinical and histopathologic features of a possible pentosan polysulfate-associated colopathy in an endoscopically and histopathologically characterized cohort and conducted a cross-sectional analysis of patients with interstitial cystitis, in which pentosan polysulfate use was associated with a diagnosis of inflammatory bowel disease (adjusted odds ratio 3.3, 95% CI 1.2–8.8). Detailed clinical phenotyping may reveal outcomes that are poorly captured using routinely collected data. At the population level, Hemida et al. used multiple cause-of-death files to trace mortality involving both opioid overdoses and cardiovascular diseases from 1999 to 2023. The age-adjusted mortality rate rose from 0.36 to 4.08 per 100,000 population (average annual percentage change 10.96%, 95% CI 8.60–13.38). Marked differences were observed in terms of sex, race, region, and rurality, indicating an overlap between the opioid epidemics and cardiovascular mortality.

## Prescription patterns and treatment trajectories in chronic disease care

While the previous two themes evaluated individual medicines, the third theme focused on prescription patterns and treatment trajectories in chronic disease care. These include regimen complexity, treatment initiation, persistence, and discontinuation in routine care.

Amrouch et al., on behalf of the Atrial Fibrillation Integrated Approach in Frail, Multimorbid and Polymedicated Older People (AFFIRMO) project, applied a reduced, operationalized set of the Screening Tool of Older People's Prescriptions and Screening Tool to Alert to Right Treatment (STOPP/START) version 2 criteria (6) to 203,042 older adults in Sweden with atrial fibrillation and at least one comorbidity. They extended the evaluation beyond oral anticoagulants to the full medication regimen and examined the associations between potentially inappropriate prescriptions and mortality, hospitalization, stroke, bleeding, and falls. Sukik et al. evaluated polypharmacy phenogroups across heart failure phenotypes in 4,902 patients in Qatar. Major, but not excessive, polypharmacy was associated with lower mortality in patients with preserved or mildly reduced ejection fraction (hazard ratios 0.62 [95% CI 0.52–0.75] and 0.70 [95% CI 0.59–0.85], respectively). In these groups, major polypharmacy largely reflects guideline-directed medical therapy. Lin et al. found a low uptake of several guideline-recommended treatments among 5,356 hospitalized patients with heart failure; angiotensin receptor-neprilysin inhibitors were used in 12.7% of the patients, sodium-glucose cotransporter 2 inhibitors in 8.1%, and quadruple therapy in 3.75%. Several of these agents were also associated with lower odds of readmission.

This theme extends beyond regimen composition to the conditions under which treatment is initiated, maintained, or discontinued. Shen et al. traced stroke prevention prescriptions among 13,072 patients with coexisting atrial fibrillation and chronic obstructive pulmonary disease in Taiwan from 2013 to 2022, and documented a marked shift from warfarin (24.0% to 6.1%) to non-vitamin K antagonist oral anticoagulants (15.4% to 65.4%). During the same period, the 1-year incidence rates of ischemic stroke and major bleeding also declined, although the study reports concurrent trends rather than an effect of the prescription shift on either outcome. Casula et al. examined the failure to initiate lipid-lowering therapy and found that 41.14% of 16,025 patients in Lombardy received no such treatment within 3 months of an atherosclerotic cardiovascular event, with older age, female sex, and polypharmacy associated with non-initiation. Campos et al. evaluated ixekizumab persistence in patients with axial spondyloarthritis at ten Spanish hospitals. At 52 weeks, 56.3% of patients remained on the treatment. The most frequently reported reason for discontinuation was primary nonresponse, followed by adverse events and secondary loss of response.

These findings demonstrate that prescriptions in chronic diseases are shaped not only by medicine count but also by regimen composition, treatment timing, and persistence over time. The arbitrary thresholds used to define polypharmacy vary widely across studies, substantially altering exposure classification and subsequent risk estimates (7). Clinically relevant questions focus on the specific agents prescribed, indications, clinical phenotypes, and how regimens are initiated, maintained, or discontinued over time. Addressing these questions requires explicit characterization of treatment regimens and trajectories, rather than relying on simple medicine counts.

## Access, reimbursement, and system-level determinants of medicine use

This theme shifts attention from treatment trajectories to the structural conditions that shape medicine use across health systems. Tang et al. used an interrupted time-series analysis to evaluate China's National Reimbursement Drug List negotiation for evolocumab and alirocumab, documenting improved availability, changes in DDDs, lower DDD costs, and a narrower gap between tertiary and secondary hospitals. Cabral et al. described antipsychotic dispensing among children and adolescents in Montevideo from 2018 to 2022, reporting increasing antipsychotic use over time, with higher utilization among providers serving populations with lower socioeconomic status. Adherence was also examined in health-system contexts that remain underrepresented in the literature. Abed et al. conducted a mixed-methods study on 405 Jordanian outpatients with multimorbidity and identified cost, polypharmacy, forgetfulness, and rural residence as barriers, together with six qualitative themes, including economic strain, religious coping, and caregiving burden. Cardona et al. analyzed 42,601 pharmacotherapeutic follow-up records of older adults in Colombia and found that inappropriate medicine use and administrative inefficiencies were prominent correlates of nonadherence, framing adherence at the system level beyond individual responsibility.

These findings demonstrate that medicine use for chronic diseases is driven not only by clinical decision-making but also by affordability, reimbursement policies, health system infrastructure, and socioeconomic contexts. For studies on adherence within these systems, medication-taking behavior should not be simplified as an individual patient trait. It should instead be conceptualized across distinct phases—initiation, implementation, and discontinuation—with persistence defined as the time from initiation to discontinuation (8). In this setting, nonadherence is not simply an individual behavior but also a product of the structural conditions under which treatment is delivered.

## Pharmacogenomics, economic evaluation, and evidence mapping

Three articles extend beyond conventional pharmacoepidemiologic study designs to pharmacogenomics, economic evaluation, and evidence mapping. Zhao et al. investigated the pharmacogenomic and epigenetic determinants of leflunomide response in 240 patients with rheumatoid arthritis and reported an interaction between a PON1 promoter methylation site (cg17330251) and the rs705379 variant, which may provide a basis for investigating the heterogeneity in treatment response. Drawing on a network meta-analysis, Yang et al. evaluated the cost-effectiveness of commercial Chinese polyherbal preparations for primary insomnia using specified willingness-to-pay thresholds. Liang et al. conducted a bibliometric mapping of two decades of research on traditional Chinese medicine, gut microbiota, and diabetes, describing the structure of the rapidly expanding but methodologically heterogeneous literature. What unifies these otherwise disparate contributions is that each still depends on a transparent account of how the data were generated, whether that account concerns the genotyping and methylation protocol, the trial network underlying an economic model, or the indexing rules behind the bibliometric map.

## Implications for study design

These studies show that the value of real-world evidence for chronic diseases does not depend on study scale alone. Rather, its value depends on whether the study design accurately captures the treatment trajectory and the process by which the data were generated. This collection illustrates this principle across a wide range of designs, from spontaneous reporting analyses and national claims cohorts to hospital-based patient data, primary survey data, and evidence synthesis. Five articles combined distinct data sources and evidence streams. Tan et al. and Spadotto et al. paired spontaneous reporting analyses with meta-analytic evidence; Jung et al. combined a clinically characterized cohort with a cross-sectional analysis; Zhu et al. combined disproportionality analysis with exploratory network pharmacology; and Yang et al. used a network meta-analysis to inform an economic model.

Triangulation enhances causal inference only when the underlying data sources are subject to biases operating in orthogonal or opposing directions. It offers limited utility when the biases align (9). The same logic explains why design choices can alter estimates, rather than simply refining them. Whether a calcium channel blocker signal for cancer persists depends on the exclusion of early follow-up, whether a tachycardia-reporting signal emerges relies on reporter stratification and subset choice, and whether polypharmacy portends harm depends on the extent to which the underlying regimen aligns with guideline-directed medical therapy. Such choices must be specified *a priori* and transparently evaluated, as each decision encodes implicit structural assumptions regarding the longitudinal relationships between exposure, indication, and outcome ascertainment.

Treatment patterns are also generated by health systems and not merely observed within them. Studies from Jordan, Colombia, and Uruguay point to system-level determinants of medicine use, including financial constraints and rural residence as barriers to adherence, administrative inefficiency in dispensing records, and provider socioeconomic levels as correlates of prescribing patterns. Therefore, the transportability of estimates from one setting to another requires explicit scrutiny of the contextual variations in patient eligibility, healthcare access, adherence metrics, and outcome ascertainment. Broader geographic representation matters not as an end but because it reveals these otherwise obscured sources of heterogeneity.

Methods for addressing these challenges are becoming increasingly available. While disproportionality analysis is straightforward to execute, it remains highly prone to overinterpretation (4, 10); several articles here illustrate specific safeguards, including active comparators, stratification by reporter type, time-to-onset profiling, and correction for multiplicity. Reporting guidance is now available for both disproportionality analyses (11) and pharmacoepidemiologic studies that use routinely collected health data (12). When data permit, target trial emulation (13), negative control outcomes (14), and preregistered analysis plans serve to make implicit assumptions explicit. In chronic disease pharmacoepidemiology, such design specifications should be increasingly regarded as part of the expected baseline standard of evidence.
